# Bitter taste receptor (TAS2R) 46 in human skeletal muscle: expression and activity

**DOI:** 10.3389/fphar.2023.1205651

**Published:** 2023-09-12

**Authors:** Maria Talmon, Erika Massara, Martina Quaregna, Marta De Battisti, Francesca Boccafoschi, Giulia Lecchi, Federico Puppo, Michele A. Bettega Cajandab, Stefano Salamone, Enrica Bovio, Renzo Boldorini, Beatrice Riva, Federica Pollastro, Luigia G. Fresu

**Affiliations:** ^1^ Department of Health Sciences, School of Medicine, University of Piemonte Orientale, Novara, Italy; ^2^ Department of Pharmaceutical Sciences, University of Piemonte Orientale, Novara, Italy

**Keywords:** bitter taste receptor, skeletal muscle, urine stem cells, urine stem cell-derived skeletal muscle cells, absinthin, calcium imaging

## Abstract

Bitter taste receptors are involved not only in taste perception but in various physiological functions as their anatomical location is not restricted to the gustatory system. We previously demonstrated expression and activity of the subtype hTAS2R46 in human airway smooth muscle and broncho-epithelial cells, and here we show its expression and functionality in human skeletal muscle cells. Three different cellular models were used: micro-dissected human skeletal tissues, human myoblasts/myotubes and human skeletal muscle cells differentiated from urine stem cells of healthy donors. We used qPCR, immunohistochemistry and immunofluorescence analysis to evaluate gene and protein hTAS2R46 expression. In order to explore receptor activity, cells were incubated with the specific bitter ligands absinthin and 3ß-hydroxydihydrocostunolide, and calcium oscillation and relaxation were evaluated by calcium imaging and collagen assay, respectively, after a cholinergic stimulus. We show, for the first time, experimentally the presence and functionality of a type 2 bitter receptor in human skeletal muscle cells. Given the tendentially protective role of the bitter receptors starting from the oral cavity and following also in the other ectopic sites, and given its expression already at the myoblast level, we hypothesize that the bitter receptor can play an important role in the development, maintenance and in the protection of muscle tissue functions.

## 1 Introduction

Bitterness perception is an innate aversive sense and is therefore a defence mechanism against potentially toxic substances. Indeed, bitter taste receptors (TAS2R) are mainly expressed in the oral cavity to protect us from ingesting anything that is potentially poisonous (Chandrashekar et al., 2000; Chaudhari and Roper, 2010). However, several *in vitro* and *in vivo* studies have investigated the extra chemoreceptive roles of TAS2Rs in extra-oral tissues, as well as their possible clinical implications (Jeruzal-Świątecka et al., 2020; Tuzim and Korolczuk, 2021; An and Liggett, 2018; Jaggupilli et al., 2017; Carey and Lee, 2019). The expression of hTAS2Rs is documented ectopically in a number of tissues, including the gut and stomach, the genitourinary system, the brain, the heart, the bone, white blood cells and the respiratory airways (Grassin-Delyle et al., 2013; Liszt et al., 2017; Rozengurt, 2006; Bloxham et al., 2020; Chen et al., 2017; Cheng et al., 2021; Welcome, 2020; Malki et al., 2015; Talmon et al., 2022a), suggesting a role beyond mere taste perception (Lu et al., 2017). For example, activation of bitter receptors in lung leads to the facilitation of foreign body removal, by inducing an increase in mucus secretion and cilia beating (Talmon et al., 2020; Sharma et al., 2022), while in the contracted airways smooth muscle cells they induce an important bronchodilation (Talmon et al., 2019; Deshpande et al., 2010). In addition to the lung, the expression and function of the hTAS2 receptor in smooth muscle has also been described in other organs, also demonstrating its potential as a pharmacological target (Manson et al., 2014; Zheng et al., 2017; Zhai et al., 2016; Sakai et al., 2016). TAS2R represents the second largest group (25 members) of chemosensory G-protein coupled receptors (GPCRs): in taste buds, the downstream signalling involves the dissociation of a-gustducin that then triggers PLCβ2 activation to cleave the phosphatidylinositol 4,5-bisphosphate (PIP2) into inositol 1,4,5-tri- phosphate (IP3) and diacylglycerol (DAG), followed by an increase in cytosolic calcium that leads to taste recognition in the brain (Ueda et al., 2003). Conversely, in ectopic sites the cytosolic calcium signalling pathway is strictly dependent not only on the tissue but also on the bitter agonist and the receptor under study (Talmon et al., 2022b; Lu et al., 2017; Wooding et al., 2021).

In previous work (Talmon et al., 2019; Talmon et al., 2020), while performing immunohistochemistry of tongue and lung sections, we serendipitously found that TAS2R46 was also located in the striae of skeletal muscle. Capitalizing on this evidence, we demonstrate for the first time both gene and protein expression of bitter taste receptor TAS2R46 in human skeletal muscle cells. Moreover, we show that its activation counteracts the increase in cytosolic calcium and consequent induced contraction of acetylcholine, leading us to hypothesize that this receptor may also have a protective role in skeletal muscle.

## 2 Materials and methods

### 2.1 Absinthin isolation

A voucher specimen of the Pancalieri chemotype of Arthemisia absinthium is kept in Novara laboratories. 1,300 g of leaves and flowers, powdered, were extracted with acetone (3 × 7.5 L) in a vertical percolator at room temperature, affording 97 g (7.5%) of a dark green syrup. The acetonic extract was dissolved into the minimal amount of acetone at 45 C and then 97 g silica gel was added (ratio extract/silica 1:1), finally the suspension was evaporated. Then absinthin was isolated according to Beauhaire et al. (1980), whose structure elucidation and purity were confirmed from 1H NMR (Supplementary Figure S1).

### 2.2 Immunohistochemistry analysis

The immunohistochemistry analysis was performed on µm-thick sections of FFPE with DAKO Autostainer (Dako) platform. After diagnostic procedures, remaining sections of tongue and locomotor skeletal muscle biopsies were baked for 30 min at 60°C, deparaffinised with xylene and rehydrated using EtOH washes of decreasing concentrations. For epitope retrieval, slides were treated in preheated citrate buffer and microwaved for 12 min at 650 W. The endogenous peroxidase activity was blocked by incubation in 3% H_2_O_2_ for 5 min. The incubation with primary antibody was performed for 1 h at RT, using polyclonal rabbit anti-human hTAS2R46 (dilution 1:1,500, OSR00173W, ThermoFisher). Subsequently, the reaction was revealed with Envision Dual Rabbit/Mouse detection system, using 3′3-diaminobenzidine tetrahydrochloride (DAB) as chromogen. The slides were counterstained with hematoxylin. When investigating the expression on the locomotor system, we used digastric, scalene and sternocleidomastoid muscle sections.

### 2.3 Cell culture

#### 2.3.1 Primary human myoblasts and myotubes

Primary myoblasts were obtained from 1 mm^3^ fragments of muscle biopsies, as previously reported (Garibaldi et al., 2016). Collection of biopsies was carried out in accordance with the policies of Sapienza University, and with The Code of Ethics of the World Medical Association (Declaration of Helsinki). An informed written consent was obtained from the volunteers. Each fragment was placed in a 60 mm dish in phosphate-buffered saline (PBS), muscle bundles were then separated longitudinally and subsequently chopped into smaller pieces. The small fragments were trypsinized for 40’ at 37°C on a magnetic stirrer and the reaction neutralized by serum. Samples were then centrifuged and the pellet, resuspended in PBS, was then plated in DMEM (Invitrogen) supplemented with 20% FBS (Gibco). Eight to 10 days after, the first myoblasts were visible. The clones, visible to the naked eye, were then isolated using 5 mm cloning rings. Myoblasts were maintained in DMEM supplemented with 10% heat-inactivated FCS (Gibco, Italy), l-glutamine 50 mg/mL (Sigma-Aldrich, Italy), penicillin 10 U/mL, streptomycin 100 mg/mL (Sigma-Aldrich, Italy), and sodium pyruvate 1 mM at 37°C, under a 5% CO_**2**_ humidified atmosphere for 6–7 days with a medium change every 24–36 h. In order to establish the myoblast cultures, we performed an immunocytochemistry assay with a desmin specific antibody (Sc-58745, Santa Cruz). For differentiation into myotubes, myoblasts were transferred for 24 h into differentiation medium consisting of DMEM with 5% horse serum and 1% penicillin-streptomycin and sodium pyruvate 1 mM and then further maintained in the same medium for a further 24 h upon plating onto glass coverslips at a concentration of 15 × 10^4^ per mL (24 mm diameter coverslips in 6 well plates) and maintained in DMEM/5% FCS supplemented with 10% heat-inactivated FBS (Gibco, Italy), l-glutamine 50 mg/mL (Sigma-Aldrich, Italy), penicillin 10 U/mL, streptomycin 100 mg/mL (Sigma-Aldrich, Italy), and sodium pyruvate 1 mM at 37°C, under a 5% CO2 humidified atmosphere. Experiments were performed at P2. Additionally, human primary skeletal muscle cells (SkMCs) were obtained from ATCC (catalog PCS-950-010). SkMCs were maintained in Mesenchymal Stem Cell Basal Medium (ATCC, catalog PCS-500-030) supplemented with Primary Skeletal Muscle Growth Kit (ATCC, catalog PCS-950-040). For differentiation into myotubes, cells were cultured 96 h with Skeletal Muscle Differentiation Tool (PCS-950-050).

#### 2.3.2 Urine-derived stem cells (USC) and differentiation to skeletal muscle cells (SkMCs)

Collection of human urine from healthy volunteers was approved by the local Ethics Committee (Comitato Etico Interaziendale Maggiore della Carità, Novara; authorization CE 190/20), and the work was carried out in accordance with The Code of Ethics of the World Medical Association (Declaration of Helsinki) and an informed written consent was obtained from the volunteers. USCs were isolated from 13 urine samples (30–300 ml) collected from 5 healthy individuals (age from 23 to 35 years old). The samples were preserved with 10% primary medium (DMEM/F12, 10% FBS, 1% penicillin-streptomycin, 2.5 μg/ml amphotericin B -ThermoFisher-, renal epithelial growth medium SingleQuot supplement–LGC Standard) for 24 h at 4°C before the isolation. USCs were isolated and differentiated as previously described (Talmon et al., 2022a). Briefly, urine samples were centrifuged (10’ at 400 g) and the pellet washed twice in washing buffer. The obtained cells were plated in a 0.1% gelatin coated-24-well plate in 500 μl of primary medium. 24, 48 and 72 h later 500 μl of primary medium were added. Then, 1.5 ml of medium were removed and 500 μl of proliferation medium were added. The half of the medium was daily changed. For differentiation into skeletal muscle cells (SkMCs), sub-confluent USCs (P2-P4) were transduced with a second-generation lentiviral vector carrying an inducible MyoD insert (LV-TRE-VP64 human MyoD-T2A-dsRedExpress2), that was a gift from Charles Gersbach (Addgene plasmid # 60629; http://n2t.net/addgene:60629; RRID:Addgene_60629) (Kabadi et al., 2015), plated on mouse collagen I-coated plates in differentiation medium and cultured for 28 days. Medium was changed daily. 72 h before experiments medium was changed with a differentiation medium without horse serum and containing 5% FBS (Gibco).

### 2.4 RNA isolation and qPCR

Total RNA was isolated by Trizol (ThermoFisher) from USCs, USC-SkMCs, primary myoblasts and myotubes, skeletal muscle biopsies from tongue and locomotor system. The amount and purity of total RNA were quantified at the spectrophotometer (Nanodrop, Thermo Fisher) by measuring the optical density at 260 and 280 nm. 1μg of total RNA was reverse-transcribed using a high-capacity SensiFAST™ cDNA Synthesis Kit (Bioline) according to the manufacturer’s instructions. For quantitative polymerase chain reaction (qPCR) gene specific primers (Supplementary Table S1) and TaqMan Expression Assay (hTAS2R46; Applied Biosystems) were used. TAS2Rs screening was performed with iTaq Universal SYBR Green Supermix (Biorad). For TaqMan assay, TaqMan Universal PCR MasterMix (2×) (without AmpErase UNG; Applied Biosystem) was used with a 7000 ABI Prism system (Applied Biosystems) was used. Glyceraldehyde-3-phosphate dehydrogenase (GAPDH) and β-glucuronidase (GUSβ) were the endogenous controls; nicotinic acetylcholine receptor subunits (nACHRa4 and nACHRa9) were used as positive control, cytokeratin 10 (CK10) as negative control.

### 2.5 Immunofluorescence

2 × 10^5^ cells were plated on 12 mm Ø glass dish, fixed in PAF 4% for 10′ and then incubated with the blocking buffer (3% BSA, 0.1% Triton X-100 in PBS) for 1 h at room temperature (RT). Cells were then incubated with polyclonal rabbit anti-human hTAS2R46 (OSR00173W, Thermo Fisher) primary antibody for 2 h at RT and then incubated for 45’ at RT in the dark with the secondary antibody goat anti-rabbit AlexaFluor 488 (Thermo Fisher). For nuclei and cytoskeleton staining DAPI and TRITC-phalloidin (Sigma-Aldrich) were added to the secondary antibody solution.

### 2.6 Lentiviral vectors production for hTAS2R46 shRNA

hTAS2R46 expression in USCs-SkMCs was silenced by lentiviral infection. Two lentiviral constructs targeting hTAS2R46 (TCRN0000014110 and TCRN0000014112) were obtained from TRCHs1.0 library (Dharmacon). Third-generation LVs were produced co-transfecting HEK293T packaging cells with plasmids pMDLg/pRRE, pMD2. VSVG, pRSV-Rev and transfer construct using the Lipofectamine 2000 (Invitrogen), as described previously (Talmon et al., 2019). USC-SkMCs were then transduced with the LV-shRNAs together and the silencing was assessed by qPCR.

### 2.7 Membrane potential analysis

For membrane potential analysis, SkMCs were loaded for 30 min at RT in the dark with a voltage-sensitive dye using the FluoVolt Membrane Potential Kit (Thermo Fisher), following manufacturer instructions, and the fluorescence were evaluated at the cyotofluorimeter (Attune NxT–Thermo Fisher). 20.000 were acquired to set basal conditions and then stimulated with absinthin (10 μM) and acetylcholine (100 μM) alone or combined. Data were expressed and analysed as delta of mean fluorescence intensity (MFI) before and after the stimulus.

### 2.8 Calcium imaging analysis

USCs and USC-SkMCs were plated on a pre-coated glass coverslip with 0.1% gelatin and collagen type I, respectively. For evaluation of Ca^2+^ fluctuations, cells were loaded with 5 µM Fura-2 a.m. (ThermoFisher) in the presence of 0.02% Pluronic-127 and 10 µM sulfinpyrazone in Krebs–Ringer buffer (KRB; 135 mM NaCl, 5 mM KCl, 0.4 mM KH_2_PO_4_, 1 mM MgSO_4_, 5.5 mM glucose, 20 mM HEPES, pH 7.4) containing 2 mM CaCl_2_ (30 min, RT). Then, cells were washed and incubated with KRB for 30 min to allow the de-esterification of Fura-2 a.m. For measurements of mitochondrial Ca^2+^, cells were loaded with Rhod-2a.m. (ThermoFisher) in the presence of 0.2% Pluronic-127 in KRB containing 2 mM CaCl_2_ (30 min, RT) and then incubated with KRB for 1 h to allow the de-esterification. The basal calcium was monitored for about 50 s and then cells were challenged with the following stimuli alone ore combined: acetylcholine (Ach, 100 μM; Sigma-Aldrich), absinthin (Abs 1, 10, 100 μM, 1 mM), cynaropicrin (10, 10 μM, 1 mM), strychnine (10, 10 μM, 1 mM), 3β-hydroxydihydrocostunolide (3-HDC, 1, 10, 100 μM, Sigma-Aldrich), H-89 dihydrochloride PKA inhibitor (10 μm, Sigma-Aldrich), ESI-09 (a pan-EPAC inhibitor, 10 μm, Sigma-Aldrich), and KB-R7943 carbamimidothioic-acid (KB, 10 μm, Sigma-Aldrich). During the experiments, coverslips were mounted into an acquisition chamber and placed on the stage of a Leica DMI6000 epifluorescent microscope equipped with S Fluor ×40/1.3 objective. Fura-2 a.m. was excited by alternating 340 nm and 380 nm using a Polychrome IV monochromator (Till Photonics, Germany), and the probe emission light was filtered through a 520/20 bandpass filter and collected by a cooled CCD camera (Hamamatsu, Japan). Rhod-2a.m. was excited at 552 nm and the fluorescence emission was recorded at 580 nm. The fluorescence signals were acquired and processed using MetaFluor software (Molecular Devices, United States). To quantify the differences in the amplitudes of Ca^2+^ transients, the Fura-2a.m. and Rhod-2a.m. fluorescences were expressed relative to the fluorescence intensity at the stimulation time (ΔF/F0).

### 2.9 Contraction assay

USC-SkMCs and primary SkMCs (400.000 cells/mL) were embedded in collagen gel (mouse tail, final concentration of 8 mg/mL) with or without acetylcholine (100 μM), absinthin (10 μM), PKA inhibitor (H89, 10 µM) and EPAC inhibitor (ESI-09, 10 μM) and seeded in triplicate for each condition in 24-well plates. After solidification, the gels were incubated in DMEM with 5% FBS and stimuli. Cells were left to grow, and area of collagen disk was measured from 1 h to 72 h after cells reached the confluence.

### 2.10 Statistical analysis

Statistical analyses were performed using GraphPad Prism 5 (California, United States). Data are presented mean ± SEM of ‘n’ independent experiments performed in triplicate. Data were analysed by one-way ANOVA. To adjust for multiple testing and to assess differences between control group and each of the treatment we have applied the Dunn’s test. A value of *p* < 0.05 was considered statistically significant.

## 3 Results

### 3.1 Expression of hTAS2R46 in human skeletal tissue

**FIGURE 1:**
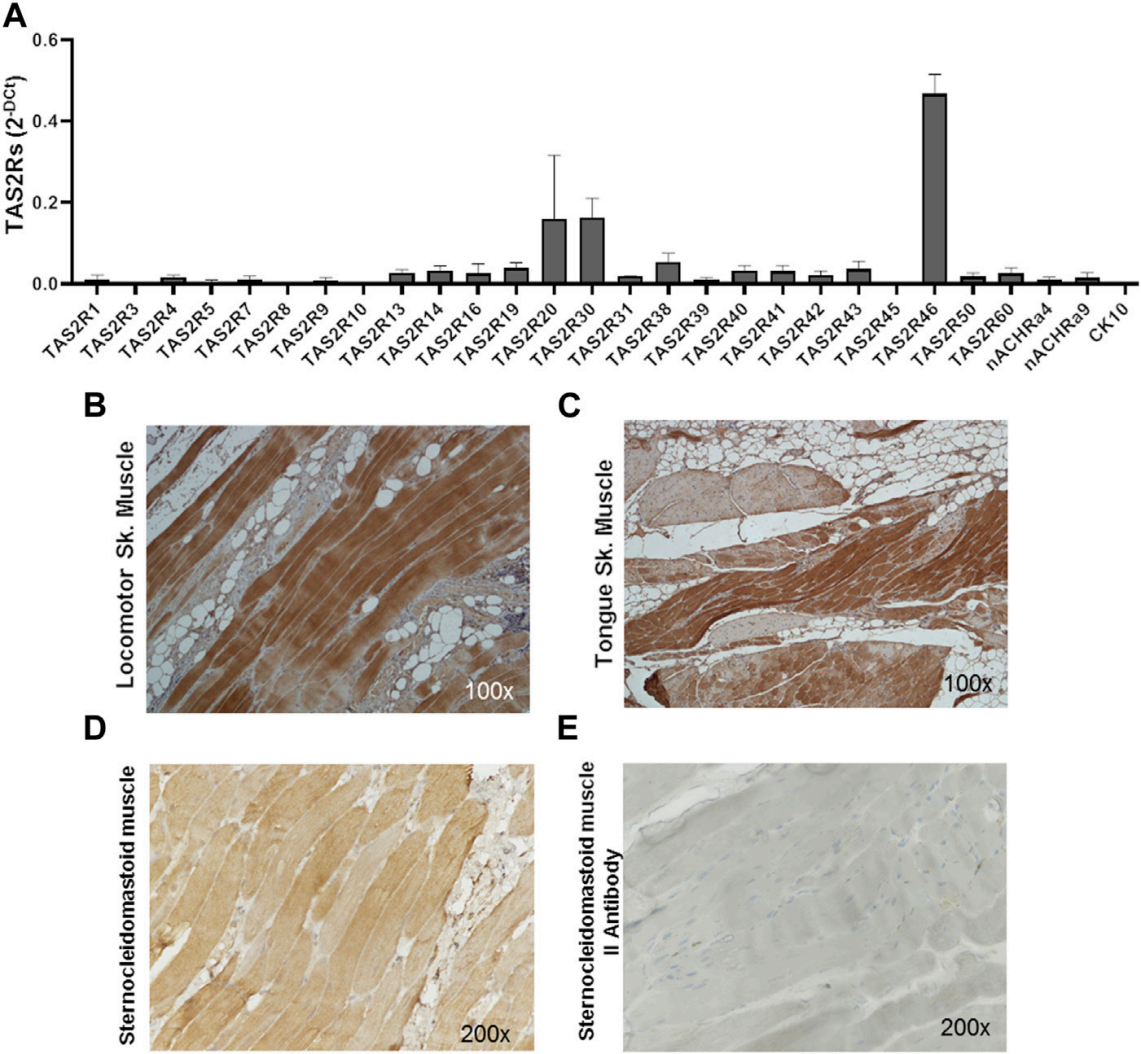
Expression of hTAS2Rs in human skeletal muscle biopsies. **(A)** qPCR analysis of hTAS2R subtypes expression in human biopsies (n = 4) of skeletal muscles (oral cavity and locomotor system). Nicotinic acetylcholine receptor subunits (nACHRa4 and nACHRa9) were used as positive control, cytokeratin 10 (CK10) as negative control. Data are expressed as 2-DCt and are means ± SEM. **(B, C)** Immunohistochemistry analysis of TAS2R46 expression on sections of locomotor system **(B)** and tongue **(C)** skeletal muscle. Magnification ×100. Positive (stained with both primary and secondary antibody, **(D)** and negative (stained with secondary antibody only, **(E)** control of immunohistochemistry analysis of section of sternoclaidomastoid skeletal muscle.

The expression of the 25 human bitter taste receptor subtypes was evaluated on skeletal muscle biopsies from the locomotor system and oral cavity. As shown by qPCR analysis (Figure 1A; Supplementary Table S1) several isoforms of the bitter taste receptor are expressed in skeletal muscle, among which TAS2R46 appears to be the most representative (Figure 1A). Immunohistochemistry analysis confirmed and highlighted that the TAS2R46 protein is well expressed all along the striated skeletal muscle fibres of both locomotor system (Figure 1B) and tongue (Figure 1C). The stain with the secondary antibody only (Figure 1E) demonstrates the specificity of the primary antibody and therefore the reliability of the receptor expression.

### 3.2 Expression of hTAS2R46 in human myoblast/myotube

**FIGURE 2:**
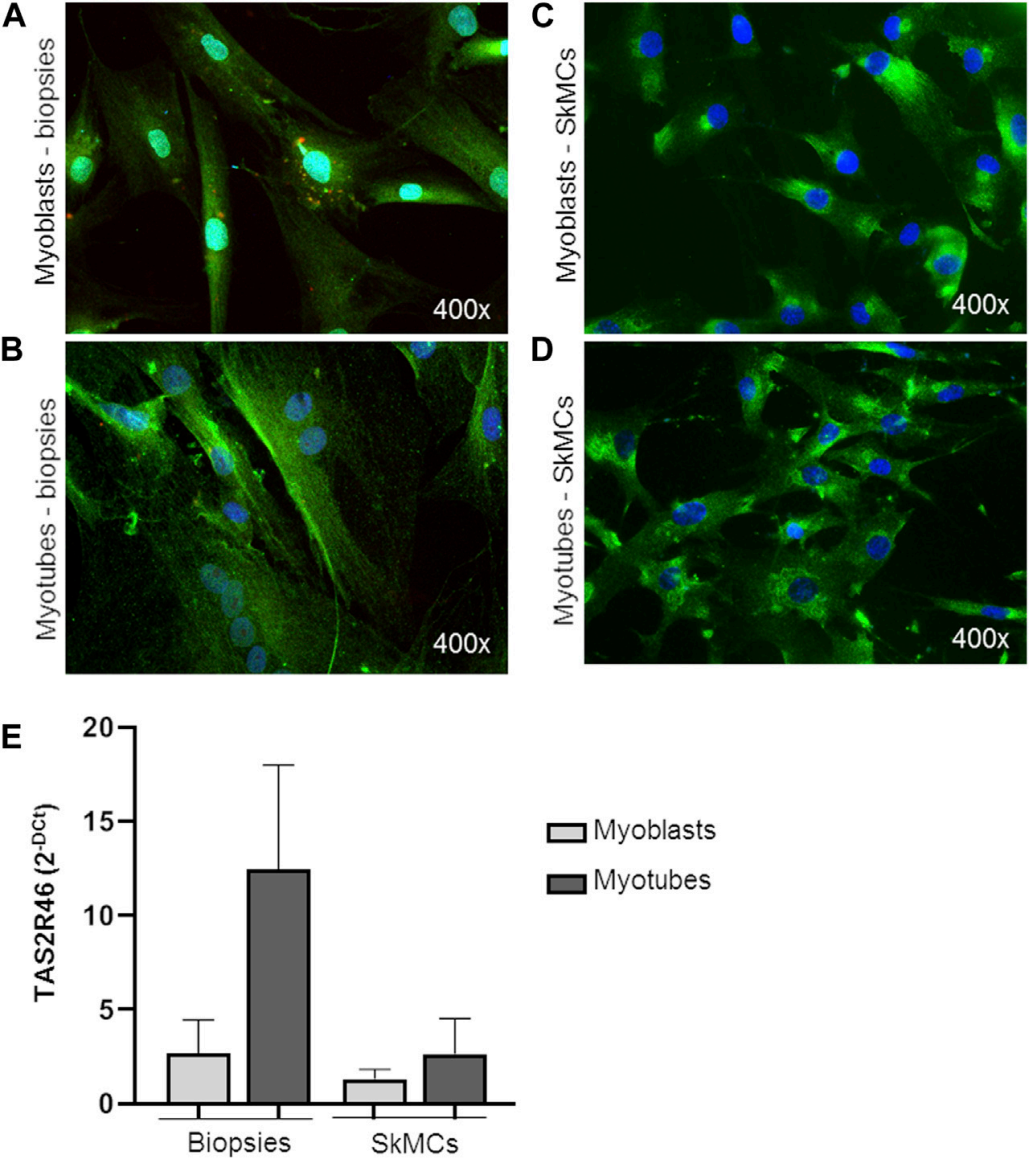
Expression of hTAS2R46 in human myoblast/myotube. **(A–D)** Immunofluorescence analysis of TAS2R46 expression in myoblasts **(A)** and myotubes **(B)** isolated from biopsies and primary SkMCs before **(C)** and after **(D)** last step of differentiation. Green: TAS2R46; Blue: Nuclei; Red. Magnification ×400. **(E)** Real time analysis of TAS2R46 expression on myoblasts and myotubes from biopsies and a primary cell line of SkMCs. Data are expressed as 2^−DCt^ and are means ± SEM of at least three independent experiments.

Both myoblasts from skeletal muscle biopsies and primary SkMC line were subcultured and differentiated into mature myotubes. hTAS2R46 expression level was evaluated in both cell types. As observed by immunofluorescence assay (Figure 2; Supplementary Figure S2) the receptor was detected in both myoblasts (Figures 2A, C) and myotubes (Figures 2B, D), with the difference that in myoblasts it was mainly localized in the perinuclear area while in the myotubes it was expressed predominantly on the cell surface. Moreover, levels of mRNA increased considerably during the differentiation process (Figure 2E; Supplementary Figure S2).

### 3.3 Expression of hTAS2R46 in USCs and USC-derived skeletal muscle cells

**FIGURE 3:**
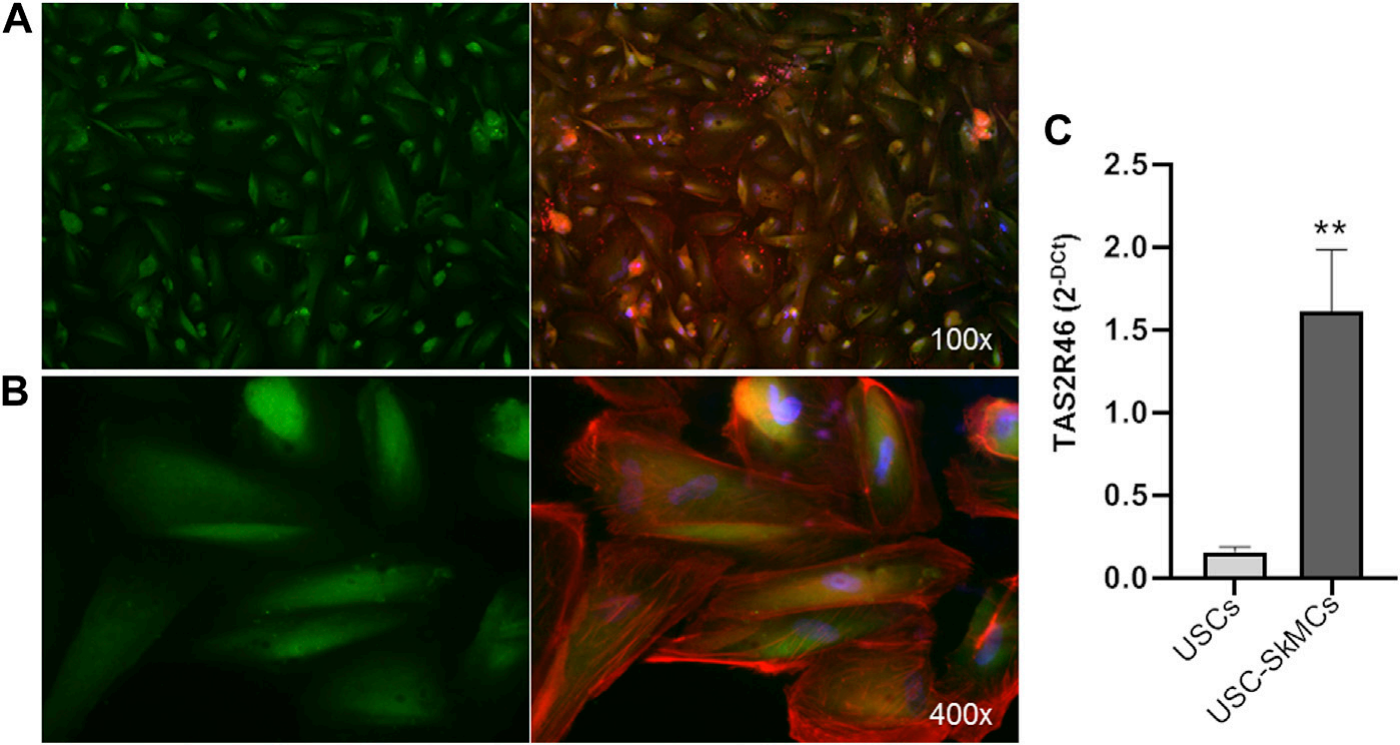
Expression of hTAS2R46 in USC-SkMCs. **(A–B)** Immunofluorescence analysis of TAS2R46 expression USC-SkMCs. Green: TAS2R46; Blue: Nuclei; Red: Phalloidin. Magnification ×100 and 400x. **(C)** Real-time PCR analysis of TAS2R46 gene transcript expression in USCs and USC-SkMCs. Data are expressed as 2^−DCt^ and are means ± SEM of five independent experiments. **p < 0.01 vs*.* USCs.

Functional characterization of primary myoblasts/myotubes is hampered by both technical and ethical issues. Therefore, we took advantage of USC-derived skeletal muscle cells (USC-SkMCs), a well characterized skeletal muscle cellular model established in our laboratory (Talmon et al., 2022b). As shown in Figure 3, expression of hTAS2R46 was demonstrated by immunofluorescence analysis also in this system (Figures 3A, B). We evaluated gene expression in both USC and USC-SkMCs and it is noteworthy the significant increase of hTAS2R46 expression after differentiation (Figure 3C; Supplementary Figure S2).

### 3.4 hTAS2R46 was functional in skeletal muscle cells

To evaluate whether hTAS2R46 was functional in skeletal muscle, we treated cells with absinthin, its most selective and potent agonist (Talmon et al., 2019), and two non-specific bitter tastants, strychnine and cynaropicrin. Absinthin was unable to induce a measurable increase of cytosolic calcium by itself at the lowest concentration tested (10 μM), although at higher concentrations a dose-dependent cytosolic calcium release could be observed (Supplementary Figure S3). The same trend and rank order of potency were observed treating cells with strychnine, while cynaropicrin gave a stronger effect reaching the maximum calcium peak at 100 μM (Supplementary Figure S3).

### 3.5 Absinthin modulates acetylcholine-induced response skeletal muscle cells

**FIGURE 4:**
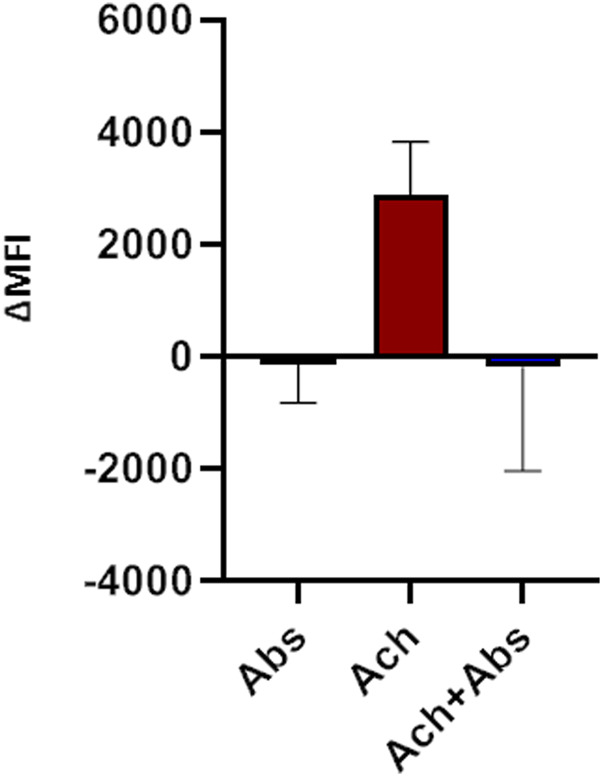
Membrane potential modulation by absinthin and acetylcholine. Human primary myotubes were charged with FluoVolt membrane dye and the variations in the fluorescence intensity were evaluated at the cytofluorimeter. Data are illustrated in histograms representing the mean ± SEM of the mean fluorescence intensity variations (ΔMFI) before and after cells stimulation with acetylcholine (100 μM) and absinthin (10 μM) alone or combined (n = 5).

**FIGURE 5:**
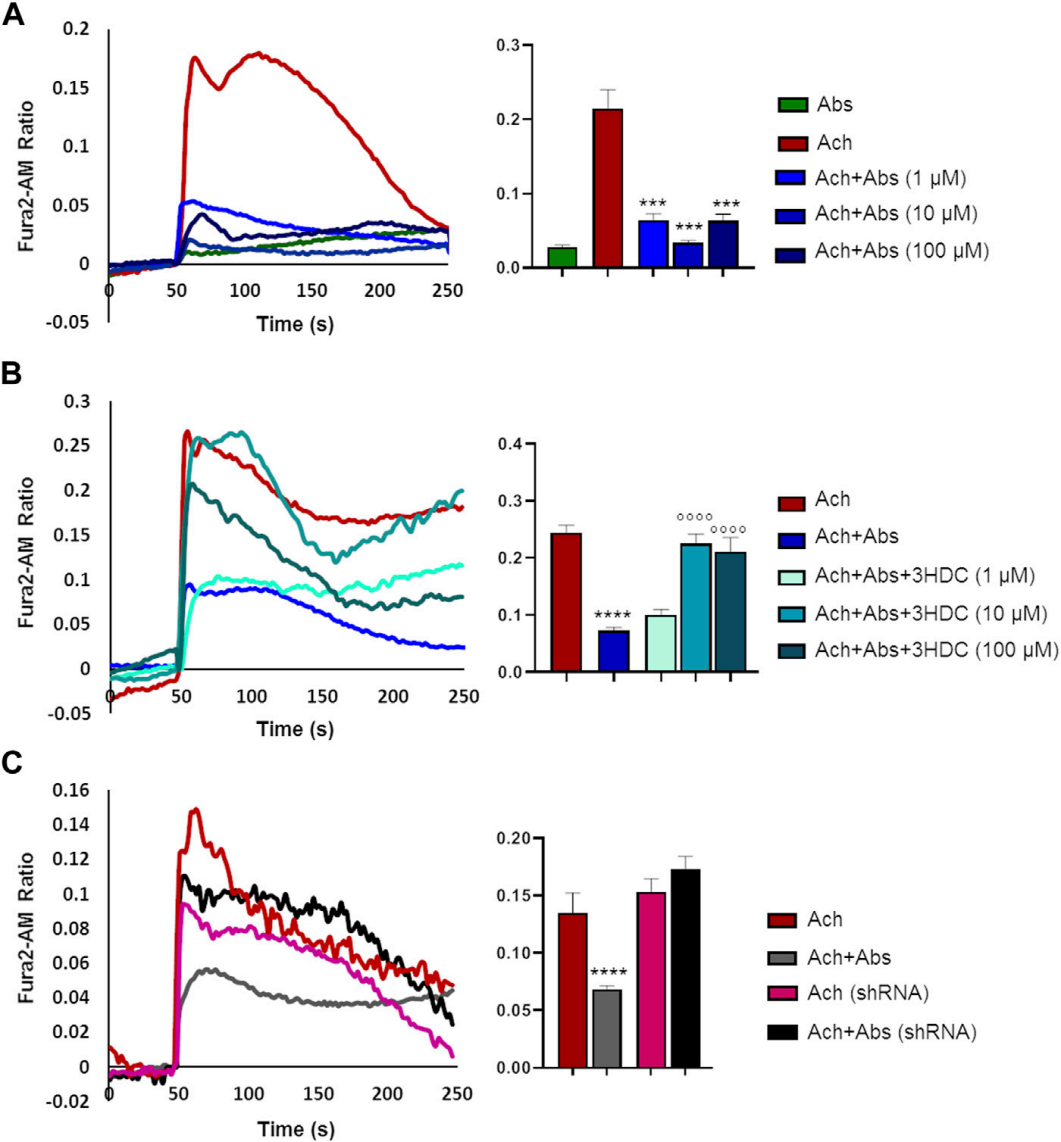
Absinthin reduces acetylcholine-induced Ca^2+^ transients in a TAS2R46-dependent manner. Data are illustrated in representative traces as well as in histogram expressing the mean ± SEM of maximum peak of cytosolic Ca2+ release of at least 32 cells in three independent experiments. **(A)** Fura-2a.m.–loaded USC-SkMCs were stimulated with acetylcholine (Ach) 100 μm and absinthin (Abs) 10 μm alone or combined. ****p < 0.001 vs*.* Ach. **(B)** USC-SkMCs were stimulated with acetylcholine (Ach) 100 μm, absinthin (Abs) 10 μm and increasing concentrations of receptor antagonist 3HDC (1, 10, 100 μm). ****p < 0.0001 vs*.* Ach; **** *p* < 0.0001 vs*.* Ach + Abs. **(C)** Fura-2a.m.-loaded USC-SkMCs silenced for hTAS2R46 expression (referred as shRNA) were stimulated with acetylcholine (Ach) 100 μm, absinthin (Abs) 10 μm alone ore combined.

We then evaluated the effect of the absinthin on the membrane potential and on calcium variations induced by one of the major pro-contractile neurotransmitters, acetylcholine (100 µM). As shown in Figure 4, acetylcholine induced a rapid membrane potential transient that was reverted by co-stimulation with absinthin. Moreover, Fura-2a.m. analysis demonstrated that acetylcholine induced a rapid increase in cytosolic calcium (Figures 5A, B) which was counteracted by absinthin, already at the lowest concentration tested (1–100 μM, Figure 5A). The effect of absinthin on acetylcholine-induced Ca^2+^-rises was strictly dependent on hTAS2R46 activation since it was reversed by 3HDC, a specific antagonist of this receptor (Brockhoff et al., 2011) (Figure 5B), and was completely abolished in USC-SkMCs silenced for TAS2R46 expression (Figure 5C; Supplementary Figure S4).

### 3.6 Absinthin modulated acetylcholine-calcium rise in a cAMP-dependent manner

**FIGURE 6:**
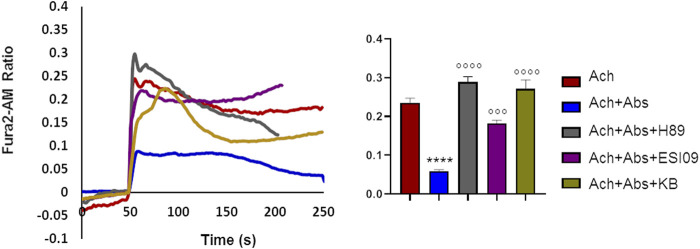
Absinthin reduces acetylcholine-induced Ca^2+^ transients via cAMP in USC-SkMCs. Data are illustrated in representative traces as well as in histogram expressing the mean ± SEM of maximum peak of cytosolic Ca^2+^ release of at least 54 cells in three independent experiments. Fura-2a.m.–loaded USC-SkMCs were stimulated with acetylcholine (Ach) 100 μm, absinthin (Abs) 1 **μ**M in presence of the PKA inhibitor H-89 (10 μM), or a pan-EPAC inhibitor (ESI-09, 10 μm) or MCU inhibitor KB-R7943 (10 μm). ****p < 0.0001 vs*.* Ach; *** *p* < 0.001**** *p* < 0.0001 vs*.* Ach + Abs.

We previously demonstrated that in airway smooth muscle cells absinthin counteracts histamine-induced calcium rise, in a similar manner to what observed with acetylcholine in skeletal muscle, stimulating mitochondrial Ca^2+^-uptake in a cAMP/EPAC dependent way (Talmon et al., 2019). To test whether this mechanism was present also in skeletal muscle, we recorded the cytosolic calcium oscillation in USC-SkMCs stimulated with acetylcholine alone or combined with absinthin, in the presence of a PKA inhibitor (H-89, 10 µM) or a specific EPAC inhibitor (ESI-09, 10 µM). As shown in Figure 6, both the co-stimulation with H-89 and ESI-09 abolished the ability of absinthin to reduce acetylcholine-induced calcium increases (Figure 6), demonstrating therefore a direct involvement of the cAMP/EPAC cascade in the downstream signalling of hTAS2R46. Since Epac1 is localized on the mitochondrial inner membrane and matrix and can control the activity of the mitochondrial calcium uniporter (MCU) (Faza et al., 2017; Wang et al., 2016), we analysed the calcium movements in USC-SkMCs co-stimulated with acetylcholine, absinthin and the MCU inhibitor KB-R7943 (carbamimidothioic acid, KB, 10 µM). Again, KB-R7043 treatment resulted in a complete inhibition of the effect of absinthin (Figure 6, yellow line and bar).

### 3.7 Absinthin induced mitochondrial calcium uptake

**FIGURE 7:**
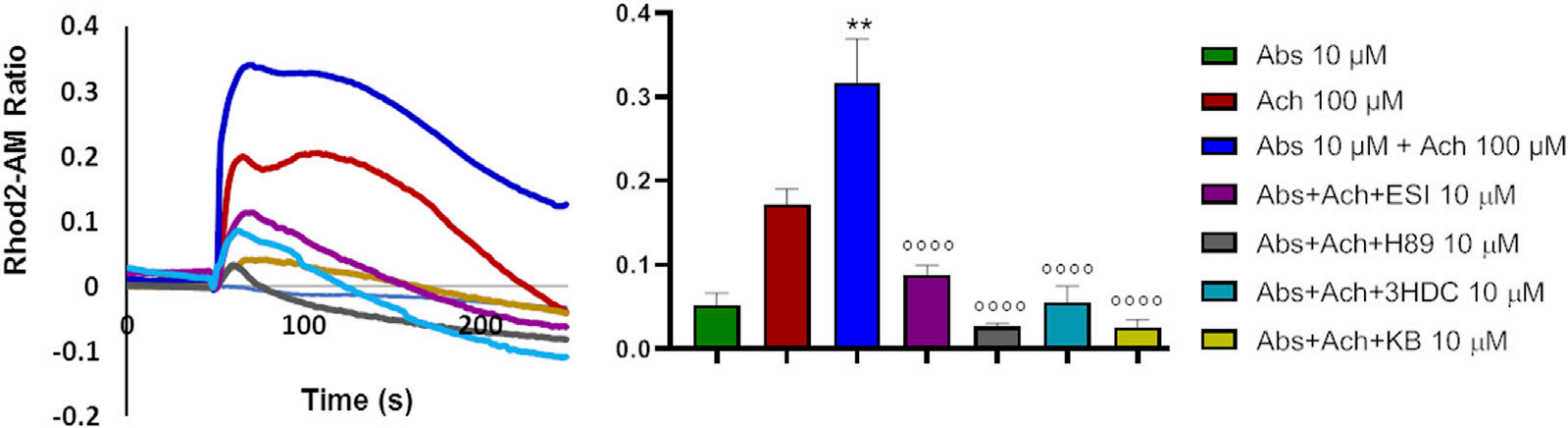
Absinthin increased mithocondrial Ca^2+^ entry. Data are illustrated in representative traces as well as in histogram expressing the mean ± SEM of maximum peak of cytosolic Ca^2+^ release of at least 43 cells in three independent experiments. Rhod-2a.m.–loaded USC-SkMCs were stimulated with acetylcholine (Ach) 100 μm, absinthin (Abs) 1 μM in presence of the antagonist 3HDC (10 μm), or the PKA inhibitor H-89 (10 μM), or the pan-EPAC inhibitor (ESI-09, 10 μm) or MCU inhibitor KB-R7943 (10 μm). **p < 0.01 vs*.* Ach; **** *p* < 0.0001 vs*.* Ach + Abs.

The above data would suggest that absinthin signals via cAMP/EPAC to the mitochondrial MCU to increase the uptake of Ca^2+^. If this were the case, it could be expected that the reduction in cytosolic calcium would be paralleled by a simultaneous increase in calcium within the mitochondria. We then evaluated mitochondrial calcium levels by Rhod2-AM analysis. As expected, acetylcholine determined a mild increase in mitochondrial calcium that was significantly augmented by the co-stimulation with absinthin (Figure 7, red and blue lines and bars). The effect was completely reverted by 3HDC, confirming that the effect is strictly dependent on hTAS2R46 (Figure 7; light blue line and bar), and by the MCU inhibitor (Figure 7; yellow line and bar). Moreover, the effect of absinthin was counteracted by the incubation with H-89 and ESI-09, confirming the involvement of cAMP in the downstream cascade and perfectly aligning the effects obtained on cytosolic Ca^2+^ to those on mitochondrial Ca^2+^.

### 3.8 Bitter agonist induces relaxation effect on USC-SkMCs

**FIGURE 8:**
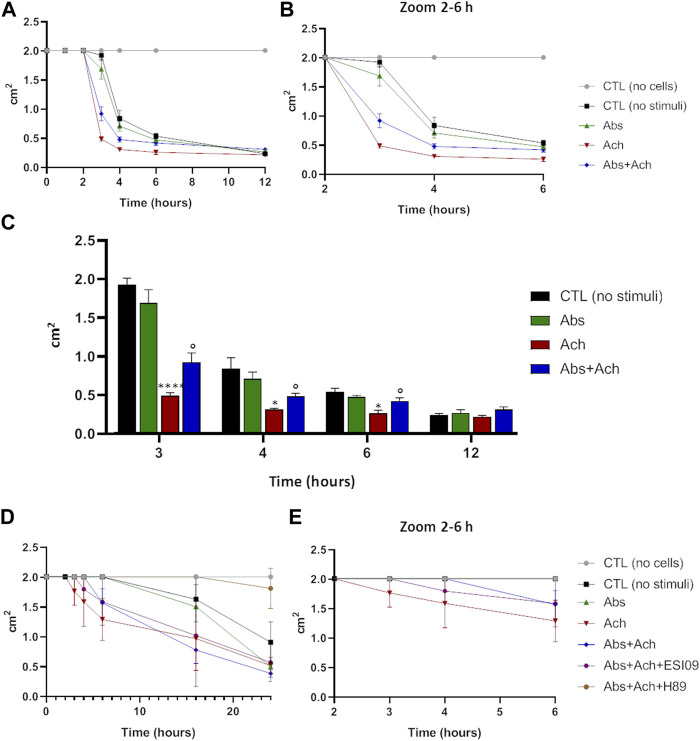
Absinthin counteracted acetylcholine-induced USC-SkMCs contraction. Cells were plated on collagen disc and treated with absinthin 10 μM, acetylcholine 100 µM. **(A)** The collagen area was measured at the indicated time point; **(B)** zoom of the time between the second and the fourth hours. **(C)** Histograms represent the mean ± SEM of collagen area (cm2) at several time points (n = 4) ****p < 0.0001; *p < 0.05 vs CTL (no stimuli); ° *p* < 0.05 vs Ach. **(D)** SkMCs were stimulated with absinthin 10 µM and acetylcholine 100 μM, also in presence of the PKA inhibitor (H89, 10 µM) and EPAC inhibitor (ESI-09); in **(E)** zoom of the time between the second and the fourth hours.

The above results, alongside providing further evidence for a novel signalling pathway that decreases Ca^2+^-signalling via increasing mitochondrial uptake, also suggest that hTAS2R46 activation might influence acetylcholine-induced contraction. We therefore tested this hypothesis in the collagen contraction assay. Skeletal muscle cells are spontaneously endowed of contraction and therefore even in absence of stimuli (Figure 8, no stimuli) it possible to measure a slight reduction of the collagen disc diameter. Interestingly**,** absinthin, alone, did not stimulate the contraction of USC-SkMCs, as demonstrated by a contraction kinetics superimposable to that of unstimulated cells (Figure 8A). As expected, acetylcholine, amplified and fastened the contraction process as early as 3 h after the addition of the neurotransmitter (Figure 8A), significantly shrinking the collagen disk, in comparison to control cells (Figure 8C). The co-stimulation with absinthin and acetylcholine slowed down the kinetic of the acetylcholine-induced contraction, and after 6 h the collagen area was still significantly larger than that of the acetylcholine-stimulated cells (Figures 8A, B). To further understand the machinery involved in the transduction signal, we performed the collagen contraction assay on primary SkMCs in the presence of both EPAC and PKA inhibitors: both compounds confirmed the ability of absinthin to contrast acetylcholine-induced contraction and strengthens the hypothesis that TAS2R46 mediates muscle dilation via cAMP (Figures 8D, E). Indeed, the EPAC inhibitor ESI-09 partially reverted absinthin effect between 3 and 4 h after contraction induction (Figure 8E).

## 4 Discussion

In the present report, using qPCR, immunohistochemistry, and immunofluorescence, we experimentally demonstrate the expression of several subtype of bitter taste receptor in human skeletal muscle cells, of which the subtype TAS2R46 showed the highest level of expression. Previously, the presence of TAS2R46 could have been hypothesized from microarrays performed on skeletal muscle (Gene Expression Omnibus), but no formal proof of its presence had been undertaken. Furthermore, we also show that TAS2R46 is functional, its activity is coupled to EPAC activation, and it antagonizes acetylcholine contraction. Because no TAS2R type had previously been demonstrated to be expressed on human skeletal muscle, we verified the expression and functionality of TAS2R46 by several different methods. While the definition of TAS2R46 as a key player in human muscle contraction is entirely novel, our results are corroborated by previous findings in rodents that suggested that bitter taste receptors might be present. Indeed, Zagorchev et al. (2019) showed that low concentrations of denatonium reduce *in vitro* the contraction of rat abdominal muscle preparations, and Kimura and Kato, (2020) the following year demonstrated the expression of different isoforms of TAS2R in murine skeletal muscle tissues and cell line, hypothesizing a role for these receptors related to metabolic functions. In human the expression of taste receptor family members has been reviewed in different non-gustatory system, both for TAS1R and TAS2R (Yamamoto and Ishimaru, 2013; Mennella et al., 2013) but so far only the former has been shown to be expressed and active in skeletal muscle. In skeletal muscle, expression of TAS1R appears to increase during differentiation, from myoblast to myocyte (Kokabu et al., 2015), and its function appears to be the sensing of nutrients, such as amino acids, and the regulation of the autophagy (Kokabu et al., 2017; Wauson et al., 2012), two critical processes for skeletal muscle function, thus suggesting that altered expression of this receptor may be involved in muscle pathologies such as sarcopenia, characterized by an incorrect autophagy (Kokabu et al., 2017). Our data show that TAS2R46 is also developmentally controlled and is expressed at higher levels in mature skeletal muscle cells. We next sought to determine the functionality of TAS2R46 by evaluating calcium activity and our results show its role in reducing contraction via a distinctive mechanism, by controlling ER/mitochondrial synapses at the MCU via a cAMP/EPAC pathway, in analogy to what previously reported in airway smooth muscle cells (Talmon et al., 2019).

As previously shown also in smooth muscle (Talmon et al., 2019), TAS2R46 activation does not disclose a direct effect alone but only in the presence of Ca^2+^-mobilizing transmitters. Indeed, we can observe a significant decrease in cytosolic calcium and a concomitant increase in mitochondrial buffering. While the Ca^2+^-handling capacity of the sarco (endo)plasmic reticulum outweighs that of mitochondria, this Ca^2+^-shuttling is significant, as shown by the Ca^2+^-traces and has repercussions on contraction. The importance of mitochondria in shaping Ca^2+^-signals has been long known (Rizzuto et al., 2012) and it is also likely that this increased Ca^2+^ also alters mitochondrial bioenergetics, although we did not directly investigate it. In skeletal muscle, the importance of mitochondrial Ca^2+^ is less characterized than in other cell types but is important to note that mutations of the proteins involved in mitochondrial Ca^2+^-transport have been very recently shown to lead to muscle dysfunction (Bulthuis, et al., 2022) providing evidence that this mechanism is highly relevant.

It would seem highly peculiar that during evolution such a sophisticated mechanism was conserved or found its place in skeletal muscle without a specific role, which we envisage being mediating fatigue or abnormal muscle contraction. Moreover, this physiological effect of absinthin in calcium modulation is so rapid that it could be interpreted as a protective response. The fact that TAS2R46 does not exclude that other bitter receptors may also be present, and their role and interplay will be of great interest to explore in the future. Indeed, we can speculate a synergy between TAS1R and TAS2R in skeletal muscle, as hypothesized by Carey et al. in cancer cells (Carey et al., 2022): on the one hand TAS1R may sensitise muscle towards nutrients, while on the other hand TAS2R may maintain calcium homeostasis avoiding cellular overwork. Therefore, our finding provides the perspective of this receptor as a target for antagonists to decrease muscle fatigue in disorders characterized by this, including dystrophies. The question arises, though, on which endogenous (bitter) compound would trigger the activation of these receptors.

## Data Availability

The original contributions presented in the study are included in the article/Supplementary Material, further inquiries can be directed to the corresponding author.
