# Supplementary material for: Bitter taste receptor (TAS2R) 46 in human skeletal muscle: expression and activity
Source: Front Pharmacol. 2023 Sep 12;14:1205651. doi: 10.3389/fphar.2023.1205651 (PMC10522851; doi:10.3389/fphar.2023.1205651)
Supplement: Supplementary file 1 [file Table1.DOCX]

Supplementary Material

# Supplementary Figures and Tables

## Supplementary Figures


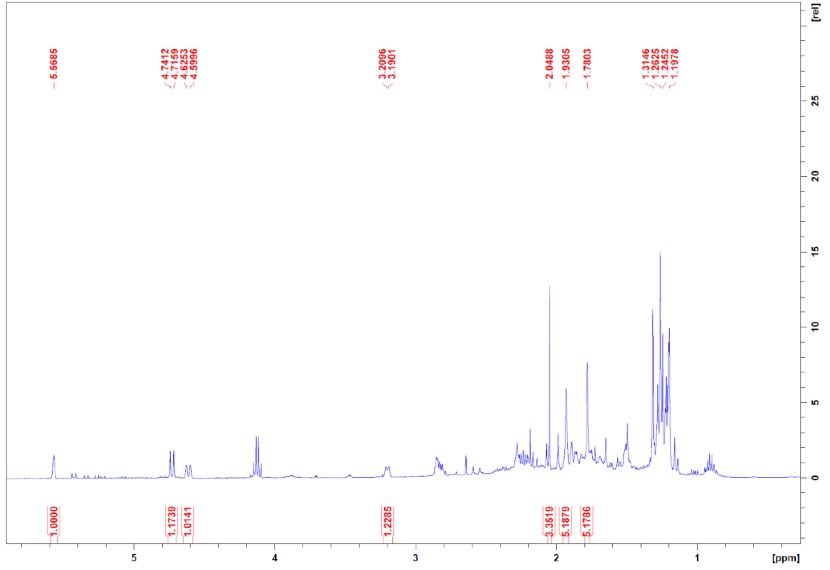


**Supplementary Figure 1.** ^1^H NMR of absinthin in CDCl_3_, 400 MHz

**Supplementary Figure 2.** Secondary controls of immunofluorescence staining for hTAS2R46 in human primary myoblasts from biopsies, SkMCs and USC-SkMCs. Immunofluorescence analysis on myoblasts omitting the polyclonal rabbit antibody anti-human TAS2R46. Green: TAS2R46; Blue: Nuclei. Magnification 200x.

**Supplementary Figure 3.** Cytosolic calcium transients induced by increasing concentrations of absinthin, strychnine and cynaropicrin. Data are illustrated in histograms of the mean ± SEM of maximum peak of cytosolic Ca^2+^  release of at least 30 cells in three independent experiment.

**Supplementary Figure 4.** hTAS2R46 silencing in USC-SkMCs. qPCR analysis (A) and immunofluorescence staining (B) of hTAS2R46 expression in USC-SkMCs and USC-SkMCs silenced for the receptor expression (referred as USC-SkMCs – shRNA).

## Supplementary Tables

**Supplementary table 1. qPCR primer sequences.**

| **Oligonucleotide** | **Sequence (5′ to 3′)** |
| --- | --- |
| hTAS2R1_for | TGTGGTGGTGAATGGCATTG |
| hTAS2R1_rev | TAAGAGAATTGCACAATTCGCAG |
| hTAS2R3_for | ACACATGATTCAGGGATAATAATGC |
| hTAS2R3_rev | TACCATCACCCTAGAAACTCTC |
| hTAS2R4_for | TACAGTGGTCAATTGCAAAACTTGG |
| hTAS2R4_rev | CTGACCTTTCCGTATTTGAAGAG |
| hTAS2R5_for | TGGTCCTCATATAACCTCATTATC |
| hTAS2R5_rev | CCAGGACCCAGAAGATACTA |
| hTAS2R7_for | GTGCTATATCCAGATGTCTATGC |
| hTAS2R7_rev | ATCCAGGAAATCACCCTGTC |
| hTAS2R8_for | AGTTATCGCCAGAATTTGTTTGATC |
| hTAS2R8_rev | GAAGACATTAAGGCAGGTGG |
| hTAS2R9_for | TGAATTGACCATAGGGATTTGGG |
| hTAS2R9_rev | AAGAGCAGCATAAAGAAGCCATC |
| hTAS2R10_for | GACTTGTAAACTGCATTGACTGTG |
| hTAS2R10_rev | GCTGGTGGCAAACCACATAC |
| hTAS2R13_for | TCAGTAAAAGAGAGCTGTCCTC |
| hTAS2R13_rev | CATAATTCTTAATCCTGTTCCAGAC |
| hTAS2R14_for | GCTTTGGCAATCTCTCGAATTAG |
| hTAS2R14_rev | TGTCCAGATATTAGTAAGCATTCTG |
| hTAS2R16_for | CCAGGCTCATACAGTTGCAT |
| hTAS2R16_rev | GAAGCGCGCTTTCATGCTT |
| hTAS2R38_for | ACAGTGATTGTGTGCTGCTG |
| hTAS2R38_rev | TGGCTTGGTAGCTGTGGTTC |
| hTAS2R39_for | TGTCGCCATTTCTCATCACCTTA |
| hTAS2R39_rev | TGCCACTTGTGGAAACTGCC |
| hTAS2R40_for | GAGTGCATCACTGGCATCCTT |
| hTAS2R40_rev | CAGCATCATCCAAATCTGTAGC |
| hTAS2R41_for | GGTTGCTGCCCTTGGATATGA |
| hTAS2R41_rev | TACTCGACCTTCTGGGCAGA |
| hTAS2R42_for | CATTTTTTTTCCTTACAAGTGGCC |
| hTAS2R42_rev | GCTTGCTGTTTCCCAGAATGAG |
| hTAS2R43_for | GCTAATGGCTTCATAGCACTGG |
| hTAS2R43_rev | TGCTGAAATGGTTGATCACTGC |
| hTAS2R31_for | GCATTGGTAAATTCCATTGAGCG |
| hTAS2R31_rev | AGCTGGATTAAACACAGTTGAATAC |
| hTAS2R45_for | CTCCTTTGCTGACCAAATTGTC |
| hTAS2R45_rev | AGTTGCTGAAATGGCCGGTTAC |
| hTAS2R46_for | GAGTTGAATCCAGCTTTTAACAG |
| hTAS2R46_rev | GGCAATCTTGAGCAAATAAAATATGC |
| hTAS2R30_for | GTTATTACTACATTGGTATGCAACTC |
| hTAS2R30_rev | GAGGCTAGTAGCAAGCCAGCT |
| hTAS2R19_for | GGTTTACTCTGGGTCATGTTATTC |
| hTAS2R19_rev | GCAAACAAAATATGCTGAGGCTAG |
| hTAS2R20_for | GCACTGATAAATTTCATTGCCTGG |
| hTAS2R20_rev | GAAGTTGGATTCAACACAGTTGAA |
| hTAS2R50_for | GGTAAATTTCATTGACTGGGTGAAGAG |
| hTAS2R50_rev | GCTGGATTCAACACAGTTAAATACCAA |
| hTAS2R60_for | CAGGCAATGGCTTCATCACTG |
| hTAS2R60_rev | TGGTCTTACCCATTACCACTG |
| nACHRa4_for | ATCCCCTCCGAGCTCATCTG |
| nACHRa4_rev | GGTCGAAGGGGAAGAAGGTG |
| nACHRa9_for | AATCATGCCGGCCTCAGAAA |
| nACHRa9_rev | CAGTGGAGGCTGTGATCAGG |
| CK-10 for | GCATGGCAACTCACATCAGG |
| CK-10 rev | CAGCCTGGCATTGTCGATCT |
